# Targeting cancer by binding iron: Dissecting cellular signaling pathways

**DOI:** 10.18632/oncotarget.4349

**Published:** 2015-06-23

**Authors:** Goldie Y.L. Lui, Zaklina Kovacevic, Vera Richardson, Angelica M. Merlot, Danuta S. Kalinowski, Des R. Richardson

**Affiliations:** ^1^ Department of Pathology and Bosch Institute, Sydney Medical School, The University of Sydney, New South Wales, Australia

**Keywords:** cancer, iron, chelators, thiosemicarbazones, signaling

## Abstract

Newer and more potent therapies are urgently needed to effectively treat advanced cancers that have developed resistance and metastasized. One such strategy is to target cancer cell iron metabolism, which is altered compared to normal cells and may facilitate their rapid proliferation. This is supported by studies reporting the anti-neoplastic activities of the clinically available iron chelators, desferrioxamine and deferasirox. More recently, ligands of the di-2-pyridylketone thiosemicarbazone (DpT) class have demonstrated potent and selective anti-proliferative activity across multiple cancer-types *in vivo*, fueling studies aimed at dissecting their molecular mechanisms of action. In the past five years alone, significant advances have been made in understanding how chelators not only modulate cellular iron metabolism, but also multiple signaling pathways implicated in tumor progression and metastasis. Herein, we discuss recent research on the targeting of iron in cancer cells, with a focus on the novel and potent DpT ligands. Several key studies have revealed that iron chelation can target the AKT, ERK, JNK, p38, STAT3, TGF-β, Wnt and autophagic pathways to subsequently inhibit cellular proliferation, the epithelial-mesenchymal transition (EMT) and metastasis. These developments emphasize that these novel therapies could be utilized clinically to effectively target cancer.

## INTRODUCTION

Cancer is estimated to have claimed the lives of more than 8 million individuals in 2012 and remains a leading cause of death worldwide [1]. Increased knowledge concerning the etiology, pathobiology and treatment of cancer has meant that some patients now have a high chance of cure. However, despite promising advances in the treatment of some cancer-types, others continue to retain dismal survival rates, such as pancreatic cancer, which has usually metastasized at the time of diagnosis and is virtually impossible to cure [2].

Although many conventional chemotherapeutics extend lives, more potent and specific therapies are required to combat advanced disease. Recent advances in cancer research have been driven by a call to understand the molecular differences between normal and neoplastic cells in order to identify cancer-specific molecular targets. This need has generated the basis for the development of the next generation of chemotherapeutics, *e.g*., trastuzumab, erlotinib, *etc*., that have entered the clinics in the past decade and target key cell signaling pathways and effectors that are deregulated in cancers [3]. However, better therapies are still urgently needed to effectively treat more aggressive tumors, particularly those that have developed resistance and metastasized. One such strategy is to target cellular iron metabolism that is known to be markedly altered in cancer cells [4–7].

Considering that most cancers are characterized by the deregulation of multiple genes and pathways, therapeutics that can target multiple molecular signaling effectors present as promising strategies for the successful treatment of cancer. Herein, we review recent research on the targeting of iron for cancer therapy, with emphasis on the novel DpT class of thiosemicarbazone iron chelators. We will discuss recent advances in understanding how these potent compounds not only target cellular iron, but also multiple signaling pathways that are implicated in tumor progression and metastasis, and how they may be integrated into the current landscape of molecular targeted therapies for cancer treatment. These and continuing developments will help to facilitate the translation of these novel therapies as a potential multi-focal strategy for cancer treatment.

## IRON AND CANCER

An excess of iron in the body is toxic due to its ability to engage in redox cycling and promote free radical formation [4]. Iron *via* the Fenton reaction can exacerbate the consequences of hydrogen peroxide production, leading to the generation of hydroxyl radicals, the most reactive chemical species in biological systems (Equation 1) [8, 9]. The superoxide ion can also participate in regenerating ferrous iron that is required for the Fenton reaction (Equation 2) [10].

(1) $$H_{2}O_{2}+Fe^{2+}\to OH^{-}+\cdot OH+Fe^{3+}$$

(2) $$O_{2}^{-}\cdot +Fe^{3+}\to O_{2}+Fe^{2+}$$

These free radicals can instigate a series of chemical reactions, leading to DNA oxidation, mitochondrial damage and the peroxidation of membrane lipids, thereby initiating DNA damage, adversely affecting cell cycle progression and promoting cell apoptosis [8]. Therefore, it is crucial that iron levels are tightly regulated in the body *via* specialized proteins and processes responsible for transporting and storing iron in a soluble and non-toxic form [11]. Failure to maintain iron homeostasis in the body is associated with numerous disease states, including cancer, cardiovascular disease, diabetes mellitus, infections and neuro-degenerative disorders [5].

### Iron metabolism

Iron from dietary sources is absorbed within the small intestine and is then transported from the duodenal enterocyte into the blood, where it binds to the iron-binding protein, transferrin [11–13]. Transferrin is present in physiological fluids, with each transferrin molecule able to bind two atoms of ferric iron with high affinity [14–16]. Transferrin plays a critical role in transporting bound iron from sites of absorption and heme degradation (*i.e*., the intestine and liver, respectively) to cells for critical functions such as erythropoiesis and storage in ferritin. The transferrin receptor 1 (TfR1) protein, which is present on the plasma membrane, is able to bind two transferrin molecules to facilitate iron uptake by cells [11, 14, 17].

**Figure 1:**
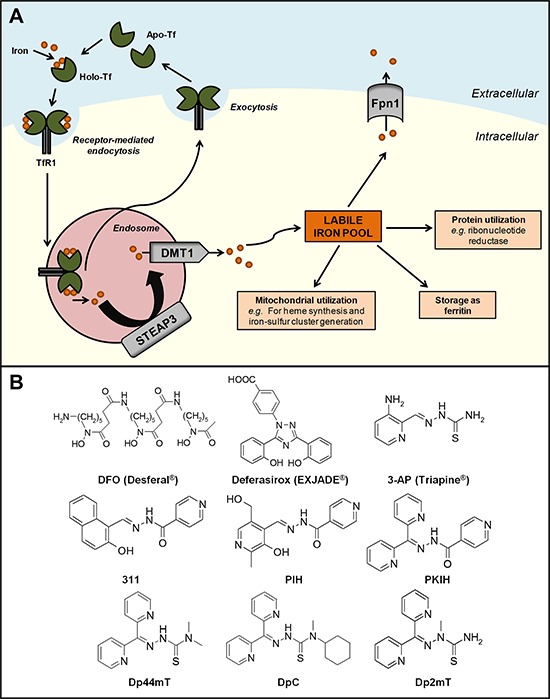
**A. Cellular iron metabolism.** Two atoms of ferric iron bind to transferrin (Tf) proteins present in the circulation to form the iron-saturated form of Tf (holo-Tf). Two molecules of holo-Tf can then bind to transferrin receptor 1 (TfR1) expressed at the cell membrane. The Tf-TfR1 complex undergoes endocytosis and is internalized into the endosome, inducing the release of Tf-bound ferric iron and reduction to its ferrous form by six-transmembrane epithelial antigen of the prostate 3 (STEAP3). Iron is then released into the cytosol *via* the divalent metal transporter 1 (DMT1) into the labile iron pool, where it can be: (i) incorporated into the active sites of proteins, such as ribonucleotide reductase; (ii) stored as cytoplasmic ferritin; (iii) utilized by the mitochondria; and (iv) exported out of the cell *via* the iron efflux pump, ferroportin 1 (Fpn1). The iron-deficient form of Tf (apo-Tf) and TfR1 molecules are subsequently recycled and returned to the cell surface. **B. Chemical structures of key chelators discussed in this review.** The structure of clinically available iron chelators, DFO and deferasirox, and anti-cancer thiosemicarbazone or PIH chelators, 3-AP and 311, respectively. The lead compounds of the first and second generation DpT thiosemicarbazones derived from PKIH analogues are shown, namely Dp44mT and DpC, respectively. Dp2mT is a structural analogue of Dp44mT that cannot bind iron and is used in studies as a negative control compound.

The transferrin-TfR1 complex is then internalized into the endosome, where the ferric iron is released from transferrin due to the acidic environment and reduced to its ferrous form, at least in erythroid cells, by the ferrireductase, six-transmembrane epithelial antigen of the prostate 3 (STEAP3; Figure 1A) [18]. The iron can then be released across the endosomal membrane into the cytosol *via* the divalent metal transporter 1 (DMT1) [19] into the labile iron pool, a poorly characterized compartment where iron is proposed to complex with low molecular weight ligands, *e.g*., citrate, ATP, sugars, *etc*. [20, 21]. However, evidence for a low molecular weight pool of cellular iron in rapidly metabolizing cells was never substantiated [22]. In fact, more recently, cellular iron chaperones have been well characterized, *i.e*., human poly (rC)-binding proteins (*i.e*., PCBP1–4), which mediate the delivery of iron to ferritin and cytosolic non-heme iron-containing enzymes, *i.e*., dioxygenases [23–25].

Cellular iron is utilized in multiple homeostatic processes. For instance, it can be: **(i)** incorporated into the active sites of proteins, *e.g*., ribonucleotide reductase (RR); **(ii)** stored in cytoplasmic ferritin; **(iii)** distributed to the mitochondria for heme synthesis, iron-sulfur cluster generation, or storage in mitochondrial ferritin; or **(iv)** exported out of the cell *via* the iron efflux pump, ferroportin 1 (Fpn1; Figure 1A) [5, 26]. Iron homeostasis is achieved, at least in part, by the iron-regulatory proteins (IRPs), which respond to intracellular iron concentrations and control protein expression at the post-transcriptional level, thereby regulating the uptake, usage and storage of iron [27, 28].

### Altered iron metabolism in cancer cells

Deregulation of iron homeostasis in cancer cells compared to normal cells has been widely reported [5, 7]. These altered iron states in malignancies can occur through changes in iron uptake, efflux and storage, which may confer an increased iron acquisition phenotype by cancer cells to mediate their rapid proliferation [5, 29].

#### Iron uptake

Numerous studies have demonstrated that cancer cells have higher TfR1 levels compared to normal cells, and that higher TfR1 expression is correlated with more advanced cancers [5, 30–32]. This reflects the higher rate of iron uptake from transferrin seen in cancer cells [33]. Furthermore, antibodies against TfR1 have been shown to inhibit tumor growth [34]. The rate limiting enzyme required for DNA synthesis requires a continual supply of iron for its assembly and activity and is also up-regulated in cancer [35]. Hence, the rates of proliferation and DNA synthesis are increased in cancer cells relative to normal cells [35].

Iron uptake is also facilitated by the endosomal metalloreductase, STEAP3, which may reduce endosomal ferric iron bound to transferrin to its ferrous form in erythroid cells [18]. In fact, STEAP has been reported to be overexpressed in prostate, colorectal, lung, and pancreatic cancers, amongst others [36, 37], indicating another aspect of altered iron metabolism that may be linked with cancer.

#### Iron efflux

Studies have reported that the expression of the iron efflux pump, Fpn1, is decreased or absent in breast cancer, prostate cancer, hepatocellular carcinoma and leukemia, although these changes were not seen in brain or esophageal cancers [38–40]. Pinnix *et al*. [39] noted that reduction in Fpn1 levels occurred concomitantly with increased levels of the peptide hormone, hepcidin, which negatively regulates Fpn1 expression by promoting its internalization and degradation [41]. Moreover, decreased Fpn1 expression in breast cancer patients was correlated with poor prognosis and metastasis, independent of other risk factors, and was proposed as a prognostic marker for this disease [39], indicating potentially important roles for the Fpn1-hepcidin regulatory axis in oncogenic signaling.

Iron in cells can also be released *via* the action of the signaling molecule, nitric oxide (NO) [42, 43]. Interestingly, NO can directly bind iron and affect a variety of intracellular proteins and metabolic pathways [11, 44–47]. Furthermore, NO generated either intracellularly (*via* inducible nitric oxide synthase; iNOS) or extracellularly has been shown to result in marked release of iron from cells [48, 49]. In fact, in contrast to the mechanism of cellular iron release mediated by Fpn1 described above, NO-dependent iron release has been demonstrated to occur *via* the multi-drug resistance protein 1 (MRP1) [49, 50].

#### Iron storage

Studies in the literature also indicate that the regulation of iron storage is altered in tumors [5]. Assessment of ferritin expression levels in cancer cells and patient tissues have yielded conflicting conclusions, with some studies reporting up-regulation of ferritin [51], while others showing down-regulation of ferritin [5, 39]. Elevated serum ferritin levels have been observed in primary lung cancer [52], advanced stage C colorectal cancer [53], and neuroblastoma patients [54], which were all correlated with poorer survival rates or prognoses.

Interestingly, ferritin expression was reportedly decreased by the proto-oncogene, *c-myc*, which often has increased expression in human malignancies, allowing an increased availability of free iron for DNA synthesis and cell growth [55]. In addition, Kakhlon *et al*. [56] showed that *HRAS*-induced down-regulation of ferritin was sufficient to increase the labile iron pool and stimulate proliferation. Thus, aberrations in ferritin levels may facilitate tumor progression.

These alterations of iron metabolism in cancer demonstrate the potential for therapeutic manipulation by iron-binding ligands. In the past few decades, this has led to the discovery and development of iron chelators with anti-tumor activity [5].

## IRON CHELATORS – FROM IRON OVERLOAD DISEASES TO CANCER TREATMENT

### Clinically approved iron chelators

Iron chelation therapy was initially designed to prevent iron-mediated toxicity in iron overload diseases such as β-thalassemia [57]. In β-thalassemia, ineffective hemoglobin synthesis necessitates repeated blood transfusions to reverse the anemic state, and together with iron absorption from the gut, this leads to iron overload, resulting in potentially fatal tissue damage [58]. Sequestering iron from cells relieves the iron burden, thereby leading to the effective treatment of the iron overload associated with β-thalassemia therapy [58, 59].

Desferrioxamine (DFO), or Desferal^®^, is a hexadentate chelator isolated from the bacteria *Streptomyces pilosus*, and binds iron in a 1:1 ratio [60] (Figure 1B). It is currently the “gold standard” chelator used for the treatment of β-thalassemia [61]. However, due to its hydrophilicity and short plasma half-life, it requires administration by long subcutaneous infusion for periods of 8–12 hours [62]. This cumbersome and expensive method, along with pain and swelling at the injection site, has led to poor patient compliance [63], and the need to develop orally-active chelators.

The potential of iron chelators for the treatment of cancer first came to light in studies assessing the effect of DFO and other iron chelators on leukemia cells in culture and also in leukemia patients [64, 65]. This was subsequently followed by studies examining neuroblastoma tumor cells [66, 67] and then clinical trials in neuroblastoma patients, which showed promising results [68]. In these early studies, DFO administered in combination with other chemotherapeutic drugs, such as cyclophosphamide, etoposide and carboplatin, resulted in 50 out of 57 patients showing partial to complete positive responses [69]. However, the reported anti-tumor activity of DFO has been inconsistent, with a number of studies failing to demonstrate marked anti-tumor effects [70, 71]. This observation is likely due to the short half-life and poor membrane permeability of this compound that markedly limits its iron chelation efficacy and anti-tumor activity [4, 72, 73].

Many synthetic iron chelators have since been designed in attempts to increase efficacy, reduce toxicity and side effects, and improve oral activity [4]. More recently, the ligand 4-[3, 5-bis-(hydroxyphenyl)-1, 2, 4-triazol-1-yl]-benzoic acid (deferasirox) was successful in clinical trials in iron-loaded patients and released by Novartis as EXJADE^®^ for the treatment of β-thalassemia as a relatively lipophilic, orally active alternative to DFO [74] (Figure 1B). Data from phase II and III trials showed that the efficacy of deferasirox was comparable to that of DFO in transfusion-dependent β-thalassemia patients [75, 76]. However, deferasirox appears to be better tolerated than DFO in both adults and children [76], and the number of serious adverse events has been relatively low [77].

In recent years, there has been growing interest in the potential of deferasirox as a potential anti-tumor agent, given that it is already clinically approved, well tolerated, and is orally administered [74]. Deferasirox has been reported to inhibit the growth of hepatoma, myeloid leukemia and mantle cell lymphoma cells [78–81]. It has also recently been demonstrated that deferasirox significantly inhibited the growth of DMS-53 lung tumor and esophageal (OE33, OE19, and OE21) tumor xenografts grown in mice, with no marked effects on biochemical and hematological indices [82, 83]. Interestingly, in a patient suffering chemotherapy-resistant acute monocytic leukemia, administration of deferasirox demonstrated a remarkable anti-leukemic effect resulting in complete remission [84]. Collectively, these studies suggest that deferasirox is a candidate chelator for chemotherapy, but further extensive studies are required to validate its clinical application.

### Thiosemicarbazone chelators

In recent years, the development of more active chelators for cancer treatment has included a range of ligands [4, 6, 85, 86]. These compounds include: 3-aminopyridine-2-carboxaldehyde thiosemicarbazone (3-AP; Triapine^®^; Figure 1B) [87], Tachypyridine [88], O-Trensox [89] and novel chelators derived from pyridoxal isonicotinoyl hydrazone (PIH; Figure 1B) [90, 91], di-2-pyridylketone isonicotinoyl hydrazone (PKIH; Figure 1B) [92] and hybrid ligands derived from aroylhydrazones and thiosemicarbazones [93]. To date, 3-AP (Triapine^®^) is the only other well characterized ligand [94] besides DFO that has reached phase I and II clinical trials specifically for the treatment of cancer [95, 96]. Administered to patients either alone or in combination with other chemotherapeutics, Triapine^®^ could successfully reduce white blood cell counts by more than 50% in leukemia patients [97]. However, clinical trials using Triapine^®^ have also reported a number of unwanted side effects including: anemia, thrombocytopenia, leucopenia, methemoglobinemia [98], neutropenia, hypoxia, hypotension, fatigue, nausea and vomiting [99]. The success of Triapine^®^ in clinical trials has been further limited by the general lack of efficacy and failure of patients to respond, resulting in termination of studies before completion [100–102]. New classes of ligands have since been developed in attempts to overcome these negative effects and improve the potency of these anti-cancer agents.

Studies examining a range of PIH analogues demonstrated that 2-hydroxyl-1-naphthylaldehyde isonicotinoyl hydrazone (311; Figure 1B), possessed marked anti-proliferative activity and greater iron chelation efficacy compared to DFO [90, 91, 103]. This anti-proliferative effect was found to be due to the ability of 311 to deplete cellular iron pools required for RR activity and other processes, as well as to affect the expression of molecules regulating cell cycle progression [94, 103, 104].

Further structure-activity studies of aroylhydrazones, such as the PKIH analogues (Figure 1B) [92], and novel aroylhydrazone/thiosemicarbazone hybrid ligands [93], led to the identification of key structural characteristics important for iron chelating efficacy and potent anti-proliferative activity. From this analysis emerged the development of the di-2-pyridylketone thiosemicarbazone (DpT) series of iron chelators, of which di-2-pyridylketone-4, 4-dimethyl-3-thiosemicarbazone (Dp44mT; Figure 1B) showed far more potent chelating efficacy and anti-proliferative activity than both DFO and 311 [105]. This activity was demonstrated *in vitro* in SK-N-MC neuroepithelioma, SK-Mel-28 melanoma and MCF-7 breast cancer cells, as well as *in vivo* using cytotoxic drug-resistant, M109 mouse lung carcinoma tumors [105]. In these studies, the specifically synthesized DpT analogue, di-2-pyridylketone 2-methyl-3-thiosemicarbazone (Dp2mT; Figure 1B), which cannot bind metals, had no significant effect on proliferation [105]. This observation demonstrated that the potent activity of the DpT series was due to its ability to specifically interact with metal ions. Comparison of the activities of Dp44mT with Triapine^®^ both *in vitro* and *in vivo* showed that Dp44mT displayed markedly and significantly greater anti-tumor activity across a range of 28 tumor cell lines, including etoposide- and vinblastine-resistant cells, indicating it could overcome resistance to other anti-tumor agents [106]. In mouse xenograft studies, Dp44mT was also more potent and effective at inhibiting tumor growth than Triapine^®^ and was less toxic [106]. The potent anti-proliferative and anti-metastatic effects of Dp44mT have also been demonstrated in other studies *in vivo* using breast [107] and hepatocellular carcinoma cells [108]. Further, Dp44mT was shown to induce apoptosis *via* the mitochondrial pathway, where decreased Bcl-2 and increased Bax expression was observed with holo-cytochrome *c* release and caspase activation [105, 109, 110]. This indicated important roles for the mitochondria in mediating the molecular events that lead to chelator-induced cell death.

Attempts to further elucidate the potent anti-proliferative effects of Dp44mT revealed two important properties, namely: (1) that it had a high lipophilicity compared to DFO, enabling it to easily permeate cell membranes; and (2) that the iron complex had an appropriate redox potential to facilitate redox activity which could lead to cytotoxicity [111]. Moreover, Dp44mT was shown to enter the lysosome and become trapped due to its ionization properties and the acidic pH of this organelle [110]. Subsequently, Dp44mT binds iron and copper to form redox-active complexes that generate cytotoxic ROS, enabling permeabilization of the lysosomal membrane and induction of apoptosis [110]. More recently, it was demonstrated that Dp44mT is a substrate of P-glycoprotein (Pgp) that is also found on lysosomal membranes, and thus, mediating lysosomal trapping of Dp44mT and enhancing cytotoxicity in multidrug-resistant cells [112]. This finding revealed a novel mechanism by which Dp44mT could overcome multidrug-resistance involving the ‘hijacking’ of the lysosomal-Pgp transport system that normally mediates resistance [112].

The observation that high, non-optimal intravenous doses of Dp44mT resulted in cardiac fibrosis in nude mice [106] led to the development of the second generation of DpT analogues [5, 109, 113]. Of this series, di-2-pyridylketone 4-cyclohexyl-4-methyl-3-thiosemicarbazone (DpC; Figure 1B) is the lead compound that is currently undergoing clinical development through Oncochel Therapeutics LLC (San Francisco, CA). Significantly, DpC has demonstrated marked and selective anti-neoplastic activity when administered orally or intravenously, without inducing the cardiotoxicity of Dp44mT [109, 113]. Moreover, DpC was more effective than Dp44mT at inhibiting the growth of PANC-1 pancreatic tumor xenografts *in vivo* [109]. Elucidation of the molecular effects of Dp44mT and DpC is ongoing and critical for understanding their potent mechanism of action.

Recent advances in our knowledge of how iron chelators can modulate key cellular signaling pathways deregulated in cancer will be discussed in the next sections. In general, and where suitable comparisons can be drawn, these studies have demonstrated that iron chelators of different classes can modulate these signaling pathways in a similar manner, indicating that the underlying iron-binding activities of these compounds are important for their observed molecular effects. Differences in the properties of chelators, such as the ability of DpT chelators to generate cytotoxic ROS, may play additional roles in modulating these pathways, and are discussed in the relevant sections.

## MODULATION OF CELLULAR SIGNALING PATHWAYS BY IRON CHELATORS

### The cell cycle

It is now clear that the anti-proliferative effects of the novel chelators discussed above are due not only to their ability to bind iron, but also to their complex effects on multiple intracellular targets. Iron chelators are known to target multiple molecules which are critical in regulating progression of the cell cycle, including RR, cyclins, cyclin-dependent kinases (CDKs), retinoblastoma protein (Rb), p53, p21^WAF1/CIP1^, p27^KIP1^, N-myc downstream regulated gene 1 (NDRG1), and apoptotic proteins [104, 114].

#### Ribonucleotide reductase

**Figure 2:**
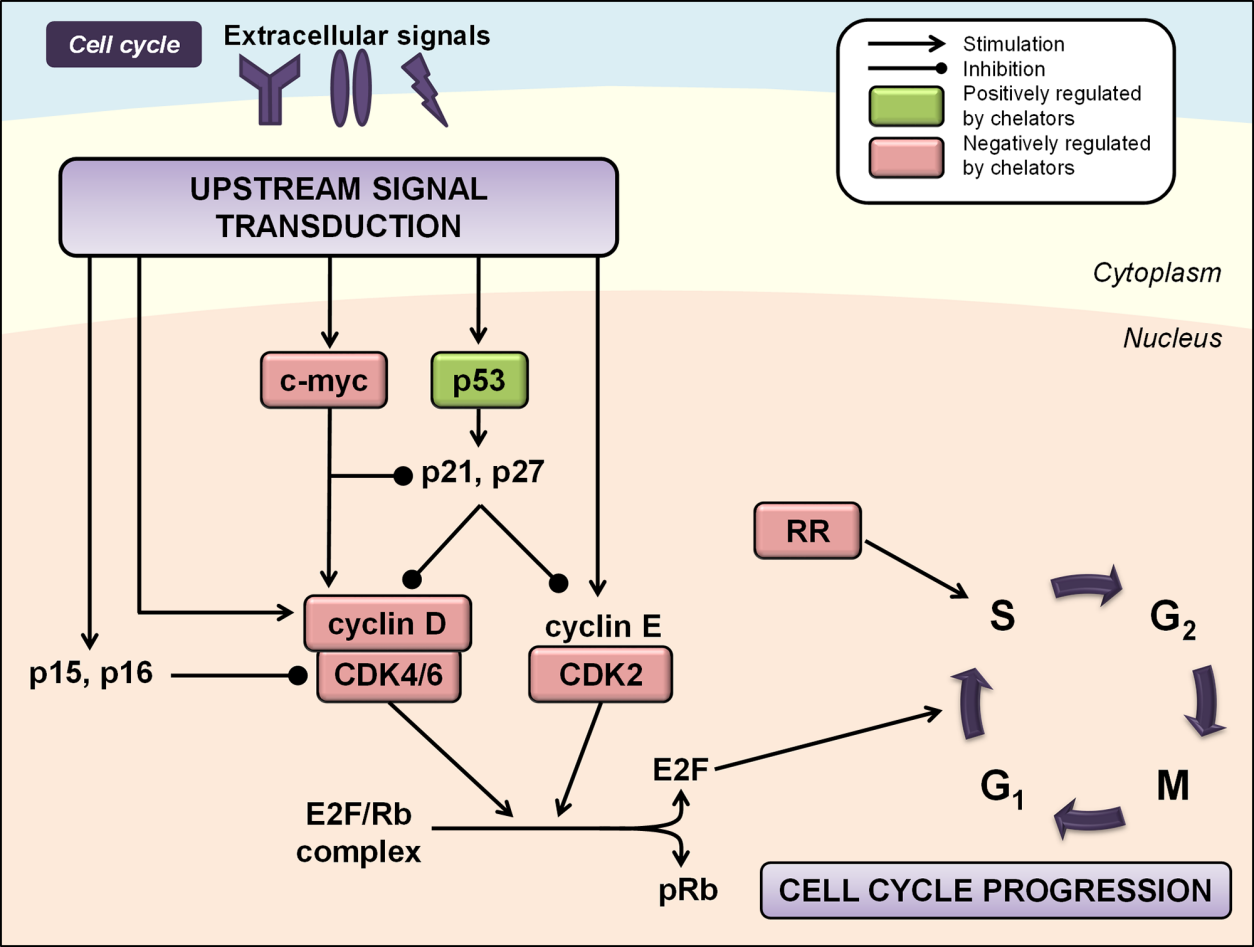
Iron chelation modulates multiple regulators of the cell cycle Regulation of cell cycle progression is mediated by the integration of multiple cues from upstream signal transduction pathways. Iron chelation has been demonstrated to modulate the activities of ribonucleotide reductase (RR), cyclin/cyclin-dependent kinase (CDK) complexes, CDK inhibitors, and other cell cycle regulators including p53 and c-myc (see text for more detail). Molecules known to be negatively regulated by iron chelators are indicated by red boxes, while molecules known to be positively regulated by iron chelators are indicated by green boxes.

RR is an enzyme responsible for catalyzing the synthesis of deoxyribonucleotides required for DNA replication, the rate limiting step of DNA synthesis, cell cycle progression and cellular repair [115]. Human RR consists of two homodimers, namely, R1, which contains the active site and binding site for allosteric modulators, and R2, which contains a di-nuclear iron center and stable tyrosyl radical per monomer crucial for enzymatic activity [116]. Furthermore, while the R2 subunit is necessary for normal DNA synthesis during the S/G_2_ phase of the cell cycle, a variant of the R2 subunit, the p53R2 subunit is known to supply dNTPs for DNA repair following DNA damage in a p53-dependent manner in G_0_/G_1_ phase cells [117]. Both the R2 and p53R2 subunits possess an iron-binding site that is critical for their enzymatic function and iron chelation is known to inhibit the activity of RR [118]. Considering increased RR activity has been associated with malignant transformation and cancer cell growth [119], the ability of iron chelators to inhibit its activity is likely to be important for their anti-cancer effects (Figure 2).

#### Cyclins and CDKs

Iron depletion by chelators has been shown to affect the expression of a number of cyclins and CDKs [104]. Iron chelation in SK-N-MC neuroepithelioma cells reduced expression of cyclins D1, D2 and D3, and to a lesser extent, cyclins A and B [104]. Inhibition of cyclin D1 expression by DFO and DpT chelators has been demonstrated in prostate and pancreatic cancer cells [120, 121], and by deferasirox in DMS-53 lung carcinoma cells [82] and mantle cell lymphoma cells [80]. This latter study also demonstrated deferasirox-mediated down-regulation of phosphorylated Rb (pRb), resulting in increased levels of the E2F/Rb complex and G_1_/S phase cell cycle arrest (Figure 2) [80]. Studies have also shown that iron chelators can decrease expression of CDK2 or CDK4, although this appears to occur in a cell-type-dependent manner and varies under different experimental conditions [94, 104, 122] (Figure 2).

#### p53

Iron chelation using DFO has been reported to up-regulate expression of the tumor suppressor, p53, at the post-transcriptional level [123]. Importantly, p53 has a critical role at the G_1_/S checkpoint and is activated in conditions of cell stress or DNA damage to either initiate repair mechanisms or induce apoptosis if damage is irreparable [124]. Incubation of cells with the chelators, DFO and 311, has also been shown to increase nuclear p53 protein and p53 DNA-binding activity (Figure 2) [125]. More recent studies have suggested that iron regulates p53 activity through direct interactions between heme and the p53 protein, which triggers nuclear export and cytosolic degradation of p53 [126]. Clearly, this latter effect is opposite to that observed after iron depletion and occurs when cells are iron replete.

#### CDK inhibitors

The CDK inhibitor, p21^WAF1/CIP1^, negatively regulates the cell cycle by binding to G_1_/S phase CDK-cyclin complexes, rendering them inactive [127]. This allows Rb to bind E2F1, thereby preventing the G_1_/S phase transition and causing cell cycle arrest. However, p21^WAF1/CIP1^ expression is required at low levels for the formation of cyclin D/CDK complexes [128], and it has also been shown to inhibit doxorubicin-induced apoptosis through repressing caspase-3 activation [129]. Iron chelation using DFO and 311 in MCF-7 breast adenocarcinoma cells has been shown to up-regulate *p21* mRNA, while decreasing the expression of p21^WAF1/CIP1^ protein [130]. MCF-7 cells have abnormally high levels of p21^WAF1/CIP1^ expression, which probably provides these cells with a survival advantage by allowing suppression of apoptosis [131]. Therefore, post-transcriptional regulation of p21^WAF1/CIP1^ by DFO and 311 confers a mechanism by which these chelators inhibit the proliferation of cells which over-express p21^WAF1/CIP1^ [130]. Incubating other cell-types, namely DMS-53 lung carcinoma and SK-N-MC cells, with the iron chelators DFO, Dp44mT, or deferasirox, increased p21^WAF1/CIP1^ expression, which would likely contribute to the anti-tumor activities of these latter two compounds *in vitro* and *in vivo* [82].

#### c-Myc

Although not classical regulators of the cell cycle, Myc proteins have profound effects on enabling cells to enter the cell cycle and accelerating cell cycle progression [132]. Myc lies at the “crossroads” of multiple signal transduction pathways, including the mitogen-activated protein kinase (MAPK), transforming growth factor-β (TGF-β), Wnt and signal transducer and activator of transcription (STAT) pathways [133, 134]. In fact, induction of *c-myc* proto-oncogene expression is required for a robust cell cycle response to mitogenic signals [135]. This likely occurs through its ability to positively regulate cyclin D/CDK4/6 activity and repress transcription of p21^WAF1/CIP1^ [136, 137]. Iron chelation using DFO has been shown to decrease c-myc expression in leukemic blood cells [138]. Further, the chelators, DFO, Dp44mT and DpC, have been recently demonstrated to reduce c-myc levels in pancreatic and prostate cancer cells (Figure 2) [121]. The mechanism mediated by these agents could at least partially involve inhibition of the STAT3 pathway and subsequent expression of its downstream targets, including cyclin D1, c-myc, and Bcl-2 (discussed in further detail below) [121].

### N-myc downstream regulated gene 1 (NDRG1)

*NDRG1* (also known as *differentiation related gene 1* or *Cap-43*) is a metastasis suppressor gene that encodes a protein with a range of significant cell functions including cell cycle regulation, differentiation, angiogenesis, maintenance of the myelin sheath of Schwann cells and mast cell maturation [139]. In fact, its pleiotropic nature is known to be cell-type-dependent [140]. While NDRG1 is widely expressed and prominent in normal cells and tissues [141], its expression is significantly lower in various cancers, including breast, colon and prostate tumors, particularly in patients with bone or lymph node metastases [142–144]. The expression of NDRG1 has also been demonstrated to have a significant inverse correlation with the Gleason grading and overall survival rate of prostate cancer patients [142], as well as the depth of tumor invasion in pancreatic adenocarcinoma patients [145]. These findings indicate an important role for NDRG1 in regulating tumor metastasis and subsequently render it an attractive target for the treatment of cancer and its spread. It is well established that intracellular iron levels regulate the expression of NDRG1, with iron-depletion markedly up-regulating NDRG1 at both the mRNA and protein levels in a variety of different cancer cell-types [146, 147]. This occurs *via* both hypoxia inducible factor 1α (HIF-1α)-dependent and -independent mechanisms [146]. Moreover, it was recently shown that translation of NDRG1 is enhanced under conditions of cellular iron depletion *via* eukaryotic initiation factor 3a (eIF3a) activity [148], which may correlate to the HIF-1α-independent mechanism previously identified [146].

NDRG1 is phosphorylated by serum and glucocorticoid-induced kinase-1 (SGK1) at Ser^330^ and Thr^346^, priming it as a substrate for glycogen synthase kinase 3 β (GSK3β), which can further phosphorylate NDRG1 at Ser^342^, Ser^352^ and Ser^362^ [149]. The ligands, DFO and Dp44mT, can increase expression of phosphorylated NDRG1 [109, 120]. However, the functional implications of NDRG1 phosphorylation have not been extensively explored in the literature and remain largely unclear. Recent studies by Murakami *et al*. [150] have suggested that in the human pancreatic cancer cell line, MIAPaCa-2, phosphorylation of NDRG1 at Ser^330^ and Thr^346^ is essential for its tumor-suppressive action on CXC chemokine expression and the nuclear factor κ-light-chain-enhancer of activated B cells (NF-κB) signaling pathway. Further, phosphorylated NDRG1 was found to be localized to centromeres and the cleavage furrow in dividing cells, implying roles for this protein in microtubule organization, the cell cycle and mitosis [151]. Of interest, recent studies demonstrated that NDRG1 is proteolytically cleaved at the *N*-terminus in the prostate cancer cell lines DU145, PC3 and LNCaP, but not in normal prostate epithelial cells [152]. This observation suggests that truncation of NDRG1 in cancer cells could abrogate its tumor growth and metastasis suppressive function.

Significant advances have been made in recent years in terms of dissecting the molecular mechanism by which NDRG1 exerts its tumor growth and metastasis suppressive activities (reviewed in [153, 154]). Importantly, NDRG1 alters the expression of a variety of transcription factors and genes involved in ribosome and protein synthesis and reduces the expression of cathepsin C, a molecule which has roles in invasion, in pancreatic cancer [147]. Additionally, whole genome gene array analysis revealed NDRG1 up-regulates *thiamine triphosphatase (Thtpa)* expression, which decreases levels of thiamine triphosphate that is required for cellular energy metabolism [147].

NDRG1 also up-regulates the CDK inhibitor, p21^WAF1/CIP1^, which may mediate its anti-tumor effects [155]. Further important roles for NDRG1 in the regulation of multiple key cell signaling pathways (*e.g*., phosphatidylinositol-3 kinase (PI3K)/AKT, MAPK and TGF-β pathways) which affect the epithelial-to-mesenchymal transition (EMT) and metastasis have also been identified [107, 109, 120, 156, 157]. These roles have been implicated in mediating, in part, the anti-tumor and anti-metastatic activities of the potent thiosemicarbazones, Dp44mT or DpC, due to the ability of these compounds to markedly up-regulate NDRG1 [107, 109, 120, 156, 157]. These findings will be discussed in further detail in the relevant upcoming sections.

### PI3K/AKT/mTOR signaling

**Figure 3:**
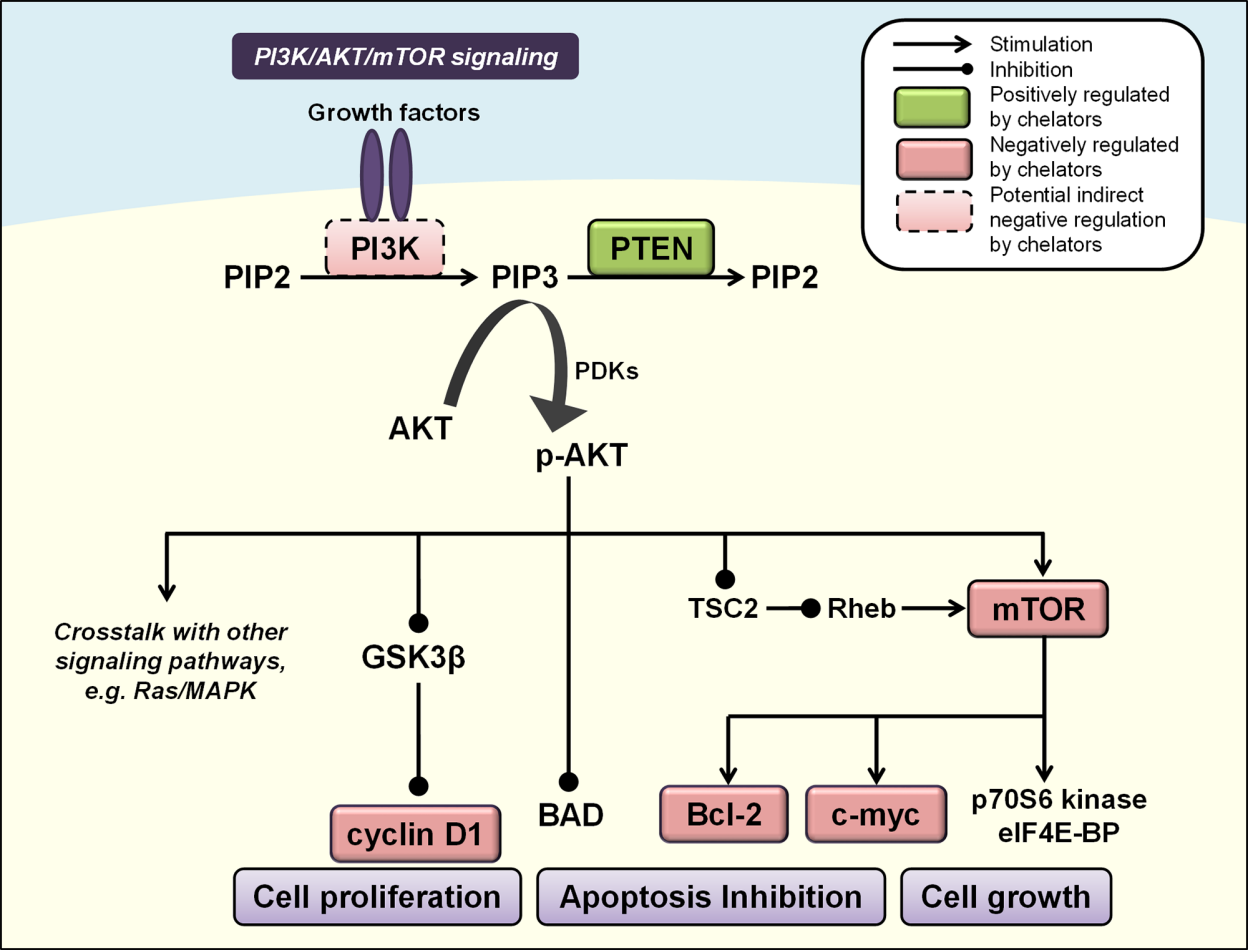
Iron chelators modulate PI3K/AKT signaling Activated PI3K catalyzes the phosphorylation of PIP2 to PIP3, leading to phosphorylation and activation of AKT (p-AKT). PIP3 levels are controlled by the phosphatase PTEN to inhibit p-AKT, which affects multiple cascades that control cell growth, proliferation and apoptosis, including GSK3β, mTOR, cyclin D1, c-myc, BAD and Bcl-2 (see text for more detail). Molecules negatively regulated by iron chelators are indicated by red boxes, and those positively regulated are indicated by green boxes. Solid boxes indicate direct evidence for modulation by iron chelators, while dashed boxes indicate potential indirect modulation, *e.g*., through chelator-mediated up-regulation of NDRG1.

The PI3K/AKT pathway has emerged in recent years as a key regulator of mammalian cell proliferation, growth and survival [158] (Figure 3). Binding of insulin or insulin-like growth factors to a receptor tyrosine kinase (RTK), such as insulin growth factor (IGF) binding to insulin growth factor receptor (IGF-R) [159], results in direct interaction between the RTK and regulatory subunits of class I PI3Ks (*e.g*., p85α, p85β, p55γ) [160, 161]. This enables the PI3K p110α/β/δ catalytic subunits to exhibit their kinase activity and catalyze the phosphorylation of phosphatidylinositol-4, 5-biphosphate (PIP2) to phosphatyidylinositol-3, 4, 5-triphosphate (PIP3) [160, 161]. The generation of PIP3 in the membrane then acts as a second messenger to activate downstream signaling effectors, including the serine/threonine kinase AKT. Specifically, PIP3 provides a docking site to recruit 3-phosphoinositide-dependent kinases (PDKs), to which AKT subsequently binds to form the phosphorylated form of AKT (p-AKT) that is active [162]. PIP3 levels are tightly controlled in mammalian cells by the action of PIP3 phosphatases, the most well characterized of which is the tumor-suppressor, phosphatase and tensin homolog deleted on chromosome 10 (PTEN; Figure 3) [163].

The activation of AKT occurs *via* phosphorylation of two residues, Ser^473^ and Thr^308^ [164], and leads to multiple signaling cascades with important consequences for cell survival, proliferation and growth [158]. PI3K/AKT signaling functions as an anti-apoptotic pathway by phosphorylating a pro-apoptotic member of the B-cell lymphoma 2 (Bcl-2) family of proteins, *i.e*., Bcl-2-associated death promoter (BAD; Figure 3), which forms a non-functional heterodimer with anti-apoptotic B-cell lymphoma-extra large (Bcl-X_L_) [165].

Activated AKT promotes cell proliferation by inhibiting GSK3β, a protein which increases cyclin D1 degradation and suppresses its expression [166, 167] (Figure 3). AKT can also regulate the activity of a number of transcription factors including cyclic-AMP response element binding protein (CREB) [168], E2F [169], NF-κB [170] and the forkhead transcription factors [171, 172], all of which can promote cell proliferation or survival.

Another major target of PI3K/AKT signaling is the serine/threonine kinase, mammalian target of rapamycin (mTOR). The activation of mTOR could occur through a number of mechanisms including direct interactions with p-AKT itself [173], or p-AKT-mediated inhibition of tuberous sclerosis 2 (TSC2), which then inhibits Rheb and leads to activation of mTOR [174–176] (Figure 3). Activation of mTOR has central roles in protein translation and cell growth, by activating p70S6 kinase and eukaryotic translation initiation factor 4E binding proteins (eIF4E-BP), which enhance mRNA recruitment to the ribosome for translation [177]. The mTOR pathway also regulates the Bcl-2 proteins involved in apoptosis and c-myc, thereby promoting cell survival, proliferation and oncogenesis [178, 179] (Figure 3).

PI3K/AKT signaling is inappropriately activated in many cancers by various mechanisms [180]. The first genetic mechanism identified that results in aberrant PI3K/AKT activation was the loss of the tumor suppressor, PTEN [180–182]. *PTEN* inactivation is seen in endometrial, central nervous system, prostate, colon, breast and liver tumors, amongst others [161]. Indeed, loss of PTEN expression has been associated with a higher Gleason grading in primary prostate tumors, with metastases displaying an accumulation of *PTEN* mutations [180].

Somatic activating mutations of the PI3K catalytic subunit, p110α, which is encoded by the *PIK3CA* gene, occur frequently in human cancers, including those of the breast, prostate, colon, and endometrium [161]. Moreover, some tumors (*e.g*., glioblastomas, colon and ovarian) also harbor activating mutations of the p85α regulatory subunit of PI3K, which is encoded by the *PIK3R1* gene, leading to constitutive PI3K-AKT signaling [183]. Furthermore, constitutively activated p-AKT is found in 30–40% of solid tumors [184], and high levels of p-AKT in prostate cancer have been associated with a poorer prognosis for patients [185].

Hyperactivation of PI3K-AKT signaling in cancer has formed the underlying basis for targeted therapies against this cascade that are currently in clinical development, including dual PI3K-mTOR inhibitors, PI3K inhibitors, AKT inhibitors, and mTOR complex catalytic site inhibitors [186]. Preclinical data suggests that such agents may be effectively used to overcome acquired resistance to therapies that target RTKs, such as trastuzumab and erlotinib, and in combination with other targeted therapies, such as mitogen-activated protein kinase kinase (MEK) inhibitors [180].

Recent studies have elucidated how iron chelators target the PI3K/AKT/PTEN pathway in prostate cancer as part of their anti-tumor activity [120]. Incubation of prostate epithelial cells and the DU145 prostate cancer cell line with DFO or Dp44mT increased expression of tumor-suppressive PTEN and anti-metastatic NDRG1 [120]. Moreover, NDRG1 over-expression attenuated oncogenic p-AKT activity and significantly increased PTEN levels [120]. NDRG1-mediated inhibition of p-AKT was also observed in PTEN-null PC3 cells [107, 120], suggesting that this effect occurred independently of PTEN.

In MIAPaCa-2 pancreatic cancer cells, NDRG1 over-expression also increased PTEN levels and decreased p-AKT and p-mTOR levels [187]. Further, expression of the PI3K regulatory subunits phospho-p85α and phospho-p55γ was significantly reduced with NDRG1 over-expression, which prevents complex formation of the PI3K p110α/β/δ catalytic subunits [187]. Given that the thiosemicarbazones, Dp44mT and DpC, markedly up-regulate NDRG1 in MIAPaCa-2 and other pancreatic cancer cell lines [109], this suggests that NDRG1 could also mediate its demonstrated anti-tumor activity in pancreatic cancer through modulation of these critical molecules in the PI3K/AKT/PTEN cascade.

Interestingly, iron chelators have been reported to increase p-AKT levels [120, 188–190]. However, this may be a short-term stress-induced response that is not characterized by its classic signaling cascade, and may be induced by these compounds before their demonstrated anti-proliferative effects overwhelm this initial pro-survival response. In fact, this elevation in p-AKT levels was not found to correlate with downstream activation of mTOR or increased cyclin D1 expression in prostate cells [120], and was consistent with previous reports that DFO inhibited cyclin D1 expression [191].

**Figure 4:**
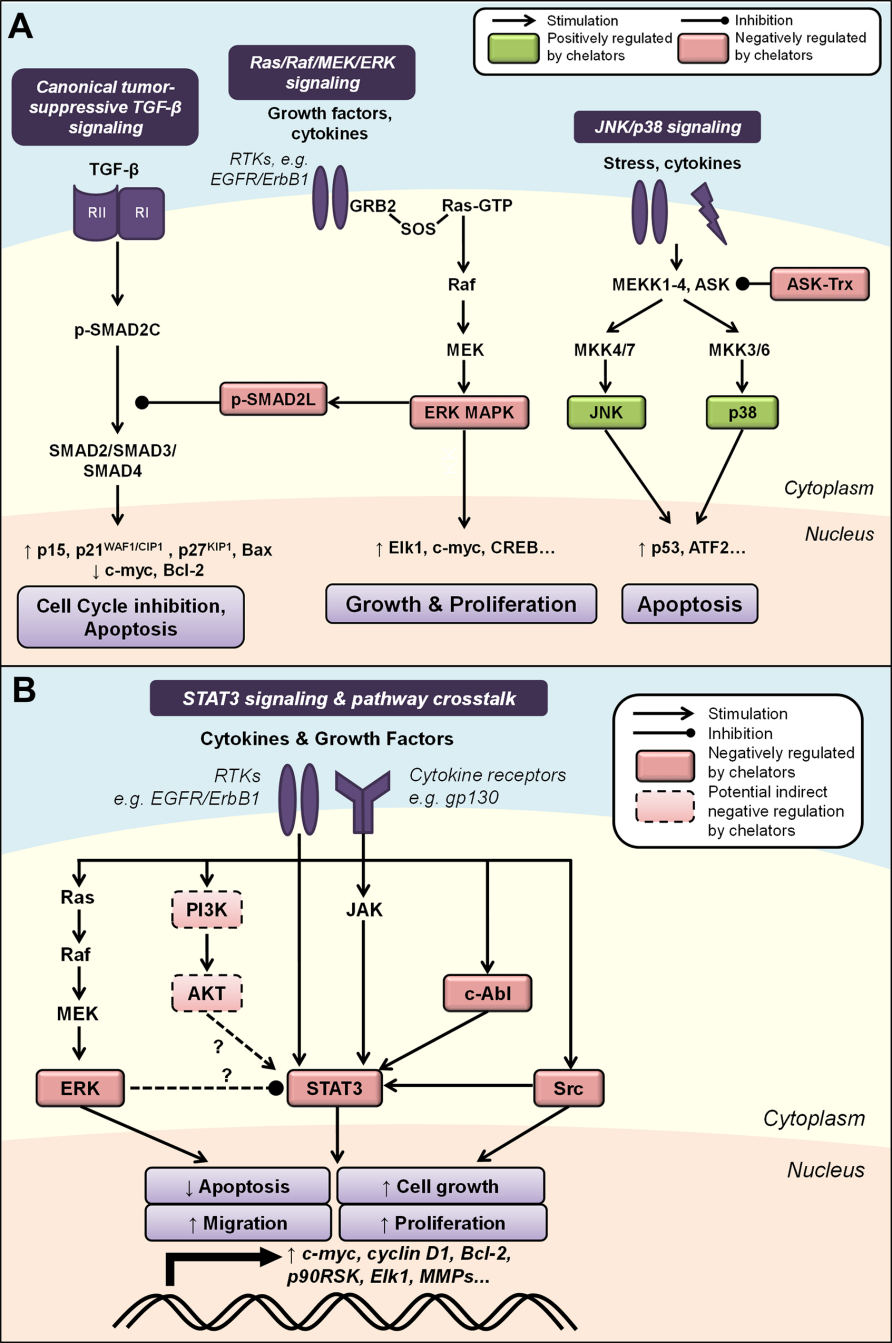
**A. Iron chelation modulates mitogen-activated protein kinase (MAPK) signaling.** Activated by growth factors and cytokines, the Ras/Raf/MEK/ERK cascade regulates cell growth and proliferation through multiple downstream effectors. ERK can also inhibit canonical TGF-β signaling by phosphorylating SMAD2 at the linker region (p-SMAD2L), preventing nuclear translocation of the SMAD2/3/4 complex and subsequent tumor-suppressive effects. The JNK and p38 pathways are two parallel MAPK cascades that are also classically activated by stress stimuli and may primarily mediate pro-apoptotic processes. Iron chelation is known to modulate the activities of ASK by dissociating its complex with thioredoxin (Trx), as well as JNK, p38, ERK and p-SMAD2L (see text for more detail). **B. Modulation of STAT3 signaling by iron chelators and integrated pathway crosstalk between the MAPK and AKT pathways.** STAT3 signaling is classically activated by cytokine receptor interactions, *e.g*., IL6/JAK, but can also be induced by growth factor receptor tyrosine kinases, *e.g*., EGFR, and non-receptor kinases, *e.g*., Src and c-Abl. Evidence also exists for potential STAT3 pathway crosstalk between the ERK and AKT pathways. Iron chelation has been demonstrated to inhibit STAT3 activity, which could be mediated by chelator-mediated effects on upstream regulators of STAT3 (see text for details). Molecules that are negatively regulated by iron chelators are indicated by red boxes, and those positively regulated are indicated by green boxes. Solid boxes indicate direct evidence for modulation by iron chelators, while dashed boxes indicate potential indirect modulation, *e.g*., through chelator-mediated up-regulation of NDRG1. Dashed arrows indicate interactions that remain to be fully characterized (see text for details).

Moreover, studies in K562 human myeloid leukemia cells have demonstrated that the orally active iron chelator, deferasirox, actually represses mTOR signaling [78]. This was observed to occur *via* enhanced expression of regulated in development and DNA damage response protein 1 (REDD1) and its downstream target, TSC2, which inhibits mTOR activity. Moreover, deferasirox repressed expression of S6 ribosomal protein and phosphorylated S6, a downstream target of mTOR [78]. Given the potential of clinically available deferasirox to be utilized as a chemotherapeutic agent [82, 83], this reveals an important mechanism by which deferasirox can modulate a key downstream mediator of oncogenic PI3K/AKT signaling, which can also integrate signals from other pathways (*e.g*., MAPK and TGF-β; see relevant sections below Figures 4A, 4B) [192, 193].

### Ras/Raf/MEK/ERK signaling

Mammalian cells possess four major MAPK protein kinase cascades [194]. These include the Ras/Raf/MEK/ERK pathway that is typically activated by growth factors and cytokines, and the c-Jun *N*-terminal kinase (JNK), p38 and ERK5 pathways that can be activated by environmental stress [194]. The Ras/Raf/MEK/ERK cascade regulates a range of important cellular processes including proliferation, survival, differentiation, migration and angiogenesis [195]. It is activated at the cell surface upon binding of ligands to RTKs such as epidermal growth factor receptor (EGFR; also known as ErbB1 or HER1), which was the first RTK to be discovered [196] (Figure 4A). Three additional family members have been subsequently identified, namely ErbB2/HER2, ErbB3/HER3 and ErbB4/HER4 [197]. The binding of a growth factor to its receptor (*e.g*., epidermal growth factor (EGF) binding to EGFR), induces an extracellular conformational change that facilitates the dimerization of two receptor monomers [198]. This enables the auto-phosphorylation of tyrosine residues in the cytoplasmic kinase domains of the receptors that creates a high-affinity binding site for the Src homology 2 (SH2) domain of growth factor receptor-bound protein 2 (GRB2), thereby recruiting it to the membrane [197]. Subsequently, the two Src homology 3 (SH3) domains of GRB2 interact with son of sevenless (SOS), which facilitates the activation of the intracellular transducer, Ras, by the exchange of GDP with GTP [199] (Figure 4A).

Ras was the first identified oncogene involved in neoplastic transformation, and is considered a key player in regulating cell growth, integrating growth factor signals with crucial effectors that relay these signals to the nucleus [200–202]. Activated RAS (RAS-GTP) subsequently recruits and activates one of its main effectors, the mitogen-activated protein kinase kinase kinase (MAPKKK, or MEKK), Raf [195]. Raf phosphorylates mitogen-activated protein kinase kinase (MAPKK, or MEK), which then phosphorylates the mitogen-activated protein kinases (MAPKs), otherwise known as extracellular signal-regulated kinases (ERKs; Figure 4A) [195]. Activated MAPKs can enter the nucleus to phosphorylate and regulate the activity of many transcription factors, including c-myc, Elk1, the O subclass member of the forkhead family of transcription factors (FOXO3), and CREB, to promote cell growth and proliferation (Figure 4A) [195]. The Ras/Raf/MEK/ERK pathway can also inhibit apoptosis by phosphorylating and inactivating pro-apoptotic Bim and BAD, thus promoting the anti-apoptotic roles of Bcl-2 and Bcl-xL [203–205]. Moreover, ERK has been implicated in the induction of cell motility and metastasis through its ability to enhance the activities of matrix metalloproteinases 2/9 (MMP-2/9) [206] and myosin light-chain kinase (MLCK) [207].

Over-expression of EGFR is observed in more than 50% of carcinomas, and ErbB2 over-expression is seen in 30% of breast cancers [208]. Mutations in Ras occur in more than 90% of pancreatic cancers, and are frequently found in non-small-cell lung carcinomas, liver cancers, melanomas and thyroid malignancies [209]. Identification of these oncogenes has led to the development of targeted therapeutics directed against critical molecules involved in the EGFR-Ras-MAPK pathway [194]. Indeed, most successful targeted therapies in the clinic are primarily directed against tyrosine kinases involved in this pathway, such as EGFR, ErbB2, BCR-ABL and KIT [210, 211].

Recently, we demonstrated that DFO and Dp44mT could regulate the Ras/Raf/MEK/ERK cascade by decreasing phosphorylation of ERK1/2 in PC-3 and DU145 prostate cancer cells [120] (Figure 4A). Dp44mT could also inhibit ERK1/2 activity in hepatocellular carcinoma cells [108]. Given that ERK is activated by a range of mitogenic stimuli and represents a critical convergence point for multiple mitogenic signaling pathways [195], the ability of iron chelators to attenuate its activation could be an important mechanism by which they exert their anti-cancer effects. Subsequent inhibition of effectors downstream of ERK by these agents have also been demonstrated, including mTOR [78] and c-myc [121].

### JNK/p38

The JNK and p38 pathways are two parallel MAPK pathways that are activated by various stress stimuli, including cytokines, ultraviolet radiation and oxidative stress [212]. These pathways participate in signaling cascades controlling cellular responses to such stimuli, and in contrast to ERKs, may primarily mediate anti-proliferative and pro-apoptotic processes [213] (Figure 4A). However, the complexity of stress-activated signaling networks means that this dichotomy is likely an oversimplification, with the effects of these MAPKs being context and cell-type dependent [214].

The JNK and p38 cascades can be activated by at least four MAPKKKs, including MEKK1-4, apoptosis signal-regulating kinase 1 (ASK1), TGF-β activated kinase 1 (TAK1) and mixed-lineage kinase (MLK) [214]. These MAPKKKs subsequently activate two distinct subgroups of MAPKKs, namely MKK4/7 and MKK3/6 that activate JNK and p38 MAP kinases, respectively [215] (Figure 4A). JNK and p38 regulate the activity of several downstream kinases and transcription factors, including p53, activating transcription factor 2 (ATF2), CREB and ETS domain-containing protein (Elk1), to affect cell transcription, proliferation and survival [215, 216]. While altered expression of the MAPK proteins, JNK and p38, are often observed in human tumor cell lines and specimens, there is little evidence that these are causally involved in tumorigenesis and tumor progression [217]. Interestingly, homozygous deletion or reduced expression of MKK4 was reported in 75% of ovarian serous carcinomas, which may contribute to development of this tumor type [218]. There is also evidence that MKK4 is mutated in pancreatic, breast, colon, lung and testis cancers, albeit at a low frequency of genetic inactivation of around 5% [219].

A role for iron depletion in mediating MAPK signaling was demonstrated in human lymphocytes, where DFO-induced apoptosis was mediated by activation of the JNK and p38 MAPKs [220] (Figure 4A). Activation of the p38 pathway was supported by phosphorylation of the upstream pathway proteins, MKK3 and MKK6, as well as the downstream targets, ATF2 and MAPK-activated protein kinase 2 (MAPKAPK2) [220].

More recent studies demonstrated that incubation of DMS-53 lung cancer cells with the chelators, DFO and Dp44mT, also increased JNK and p38 phosphorylation, resulting in increased phosphorylation of the downstream targets, p53 and ATF2 [221] (Figure 4A). Iron supplementation experiments indicated that the ability of DFO and Dp44mT to bind iron were crucial for their effects on JNK activation, and that the ability of Dp44mT to promote reactive oxygen species (ROS) generation also contributed to this effect [221]. DFO- and Dp44mT-induced JNK activation was found to be mediated by Trx oxidation following iron chelation, causing dissociation of the ASK1-Trx complex that normally inhibits ASK1 kinase activity and subsequent ASK1-dependent apoptosis [221, 222].

However, studies in other cell-types have reported some variable effects of chelators on MAPK signaling pathways. For instance, using immortalized human oral keratinocytes and the head and neck squamous cell carcinoma HN4 cell line, incubation with DFO resulted in increased levels of phosphorylated ERK1/2 and p38 MAPKs, but with no effect on JNK activation [223]. Moreover, it was shown that activation of ERK and p38 were key effectors mediating downstream apoptotic signaling through the caspase cascade and mitochondrial release of cytochrome *c* [223]. DFO also activated ERK, p38, and JNK in HL-60 human myeloid leukemia cells that was accompanied by a similar activation of the apoptotic cascade [224].

The observation that DFO increases phosphorylation of ERK in this latter investigation is in contrast to our previous study using prostate cancer cells that demonstrated suppressed phosphorylated ERK levels in response to DFO and Dp44mT [120]. This may indicate potential cell-type differences in the regulation of MAPK signaling and the complexities involved in cellular pathway regulation by iron depletion. However, the reported effects of iron chelation on activating p38 appear to be more consistent, and it has been suggested that p38 functions as a tumor suppressor since inhibition of p38 function plays an important role in Ras-induced transformation [225]. Moreover, p38 MAPK was found to be a major mediator of DFO-induced apoptosis over other MAPKs [224], suggesting that the ability of iron chelators to modulate this MAPK may be critical for their cytotoxic effects.

### Canonical tumor-suppressive TGF-β signaling

The TGF-β pathway is activated when the cytokine TGF-β binds to the TGF-β receptor II (TGF-βRII) subunit expressed on cell membranes [226]. This recruits the TGF-β receptor I (TGF-βRI) subunit to the complex and causes phosphorylation of serine and threonine residues on TGF-βRI, which activates its kinase activity [226]. The receptor SMADs, SMAD2 and SMAD3, which are normally anchored by the cytoskeletal protein filamin A, are translocated to the active TGF-βRI/II complex where they are also phosphorylated at the *C*-termini at Ser^465/467^ (p-SMAD2C) and Ser^423/425^ [227, 228] (Figure 4A). This results in the formation of heteromeric complexes with cytosolic SMAD4 [229], which then translocates to the nucleus, binds to SMAD-binding elements (SBE) and thereby regulates TGF-β-responsive gene expression [229] (Figure 4A).

The canonical tumor-suppressive actions of TGF-β are mediated by its effects on the cell cycle, through the up-regulation of the CDK inhibitors, p15, p21^WAF1/CIP1^ and p27^KIP1^, as well as down-regulation of c-myc [230]. TGF-β also promotes apoptosis through up-regulation of pro-apoptotic Bax, increased activation of effector caspases, and down-regulation of anti-apoptotic Bcl-2 [231]. TGF-β has been shown to cause significant reduction in proliferation and induction of cell death in human prostatic epithelial cells [232] and some prostate cancer cell lines [233].

The level of TGF-β/SMAD signaling in cells has been reported to be negatively regulated by the Ras/Raf/MEK/ERK cascade, which is induced by EGF receptor signaling [234]. Specifically, Ras signaling inhibits the TGF-β-induced nuclear accumulation of the SMAD complex by phosphorylating SMAD2 at the linker region (p-SMAD2L), which is distinct from the TGF-β receptor phosphorylation site [234] (Figure 4A). In normal epithelial cells, TGF-β potently overcomes the proliferative effects of Ras-activating factors [235], suggesting Ras-mediated phosphorylation of SMAD2 serves to adjust the level of TGF-β/SMAD signaling according to the level of Ras activity in these cells. However, in cancer cells where Ras is hyper-activated by oncogenic mutations, this same mechanism may act to silence the tumor-suppressive functions of TGF-β/SMAD signaling and consequently promote tumor progression [234]. This suggests that in Ras-transformed cancer cells, inhibiting the phosphorylation of SMAD2 at the linker region may be a worthy therapeutic strategy to consider.

As previously mentioned, DFO and Dp44mT can attenuate phosphorylation of ERK1/2 in PC-3 and DU145 prostate cancer cells [120]. This study also demonstrated that these compounds could decrease levels of p-SMAD2L. Conversely, no changes were observed in the phosphorylation of SMAD2 at the *C*-terminal region (p-SMAD2C) that is regulated by TGF-β activation in these cells [120]. Thus, taken together, DFO and Dp44mT could suppress ERK1/2 to subsequently inhibit the oncogenic effects of SMAD2 phosphorylated at the linker region. This effect could modulate canonical tumor suppressive TGF-β signaling by overall promoting the effects of *C*-terminal-phosphorylated SMAD2 (Figure 4A).

### STAT3 signaling

The STAT family consists of seven proteins that transduce extracellular signals to regulate genes involved in cell growth, survival and differentiation [236]. Of these, STAT3 is the most well characterized and has been linked to tumor progression [236]. STAT3 signaling is classically known to be activated by upstream cytokine receptor interactions, such as those resulting from interleukins and interferons activating the Janus tyrosine kinases (JAKs) [237]. However, STAT3 signaling can also be induced by growth factor RTKs (*e.g*., EGFR) and non-receptor kinases (*e.g*., Src, c-Abl) [238–240] (Figure 4B). Activation of STAT3 results in its phosphorylation at Tyr^705^ (p-STAT3), leading to dimer formation, nuclear translocation, binding to STAT3-specific DNA-binding elements and transcription of target genes [240].

The wide and varied cellular effects of STAT3 signaling include the promotion of cell cycle progression by up-regulating cyclin D1, c-myc and pim-1, and suppression of apoptosis through Bcl-2, myeloid leukemia cell differentiation protein 1 (Mcl-1) and cellular inhibitor of apoptosis 2 (c-IAP2) expression [236, 241] (Figure 4B). STAT3 has also been shown to repress p53 expression, with blockade of STAT3 in cancer cells leading to p53-mediated apoptosis [242]. Several lines of evidence also implicate STAT3 in tumor cell invasion, migration and immune evasion as well as the EMT by promoting Rho GTPase-regulated cell migration and regulating the expression of MMPs, E-cadherin, zinc-finger E-box-binding (ZEB) and vimentin, amongst others [236, 243–245].

Cancers of the prostate, pancreas and breast, as well as leukemias and melanomas, have all been reported to display constitutive activation of STAT3 [246, 247]. This has directed numerous recent efforts to develop new therapies targeting the STAT3 pathway, including direct inhibitors of STAT3, IL-6 receptor antagonists, antisense strategies and decoy phosphopeptides [248, 249].

Recently, we demonstrated that iron chelators regulate the STAT3 pathway in pancreatic and prostate cancer cells through inhibition of constitutive and IL-6-induced STAT3 activation [121] (Figure 4B). The chelators, DFO, Dp44mT and DpC, inhibited constitutive phosphorylation of STAT3, dimerization, binding of STAT3 to its target DNA sequence, and the expression of STAT3 targets (*i.e*., c-myc, cyclin D1 and Bcl-2) [121]. This may be, in part, mediated by the ability of these compounds to decrease activation of the upstream kinases, Src and c-Abl, that are known to promote STAT3 signaling [121] (Figure 4B). Further, the redox-active Dp44mT- and DpC-iron complexes also inhibited STAT3 activation and this could be prevented by the antioxidant, *N*-acetylcysteine (NAC), indicating that ROS generation by these compounds is involved in their STAT3-inhibitory properties [121]. Both Dp44mT and DpC could also inhibit IL-6-induced phosphorylation and nuclear translocation of STAT3, which has been implicated in tumor progression [121, 248]. These findings were reiterated *in vivo* in a PANC-1 tumor xenograft model where Dp44mT- or DpC-treated tumors displayed significantly lower staining for p-STAT3 and STAT3 compared to tumors from vehicle control-treated mice [121]. Similar findings were also observed in studies examining hepatocellular carcinoma cell lines, where Dp44mT suppressed p-STAT3 levels, possibly through up-regulation of the NDRG family member, NDRG2 [108].

Interestingly, serine phosphorylation of STAT3 by ERK has been reported to negatively modulate STAT3 tyrosine phosphorylation and activity [250, 251], while other studies implicate the MEK/ERK cascade in positively mediating STAT3 activity [252–254]. The AKT/PTEN pathway has also been reported to regulate STAT3 [255, 256], and it was recently suggested that STAT3 occupies a central role in integrating pathway crosstalk between the MAPK and AKT pathways [257]. Given these interactions, it is possible that the effects of iron chelators on the STAT3 pathway are, at least in part, influenced by their modulation of the ERK and AKT pathways (Figure 4B). Indeed, more detailed characterization of the complex crosstalk between these and potentially other pathways that mediate the anti-neoplastic activities of iron chelators is required.

### The EMT

**Figure 5:**
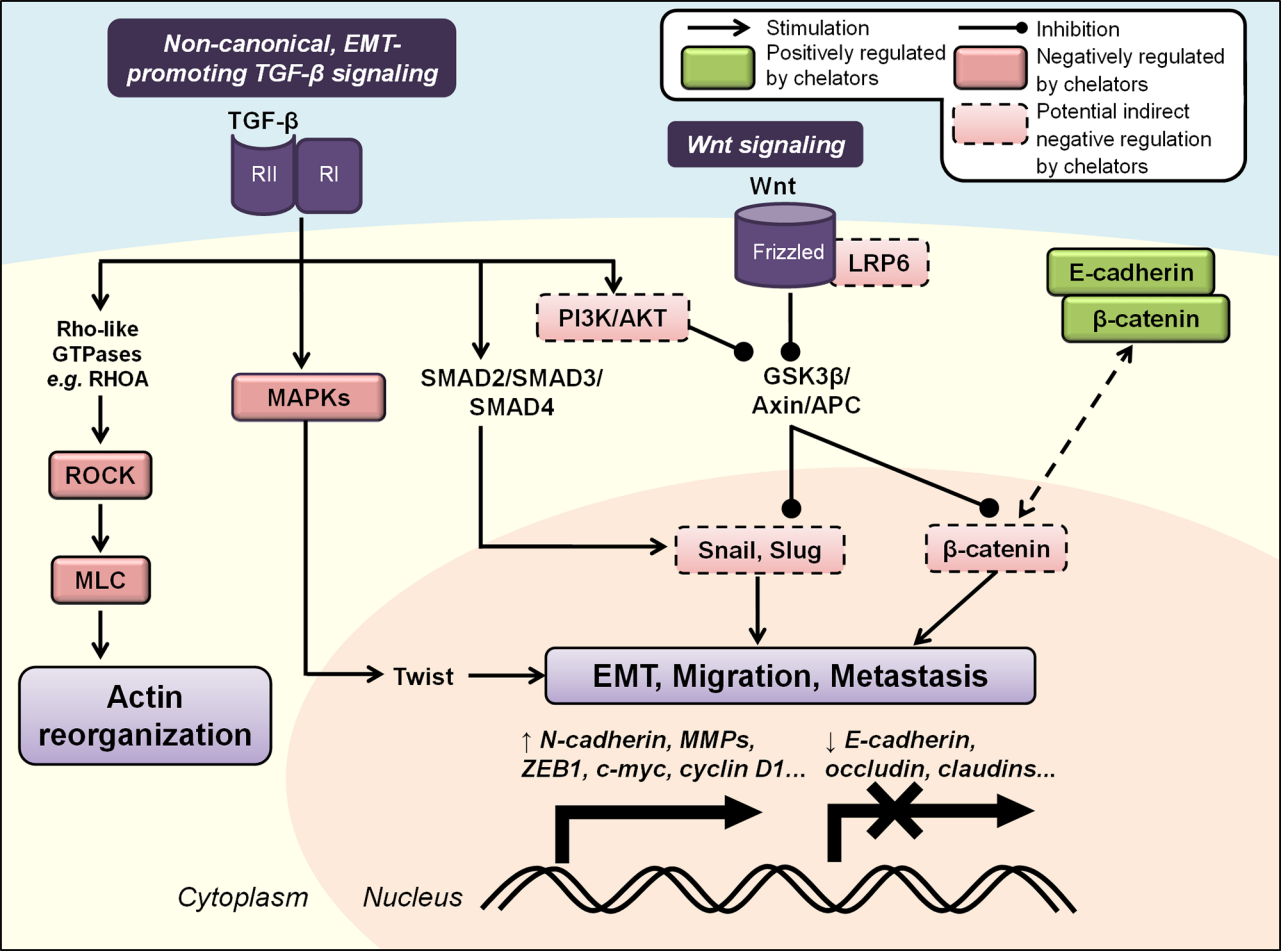
Iron chelators regulate the epithelial-to-mesenchymal transition (EMT) and molecular motors involved in cancer cell migration and metastasis Non-canonical activation of TGF-β signaling promotes tumor progression, EMT and metastasis *via* SMAD, MAPK and PI3K/AKT pathways that up-regulate mesenchymal-associated transcription factors (*e.g*., Snail, Slug, Twist and ZEB) and repress E-cadherin expression. The Wnt signaling pathway also regulates EMT through its inhibition of GSK3β-mediated degradation of β-catenin, Snail and Slug. TGF-β can also activate Rho/ROCK/MLC signaling to drive dynamic actin reorganization required for metastasis. Iron chelators have been demonstrated to inhibit the EMT and metastasis by maintaining expression of E-cadherin and β-catenin at cell membranes, and may also mediate these effects through regulation of other upstream cascades (see text for more details). Molecules negatively regulated by iron chelators are indicated by red boxes, and those positively regulated are indicated by green boxes. Solid boxes indicate direct evidence for modulation by iron chelators, while dashed boxes indicate potential indirect modulation, *e.g*., through chelator-mediated up-regulation of NDRG1.

The EMT refers to the transient differentiation process whereby epithelial cells lose their polarity and cell-cell junctions, reorganize their cytoskeleton, and induce signaling changes that reprogram gene expression and cell shape [258]. While EMT plays important roles during embryogenesis and wound healing, its underlying processes are reactivated in the pathological processes of fibrogenesis and tumorigenesis [258]. Repression of the epithelial phenotype and activation of the mesenchymal phenotype involves changes in the gene expression of many molecules, including the master transcription factor regulators, Snail, Slug, Twist, and ZEB [258] (Figure 5). These proteins often regulate the expression of each other as well as other target genes, including the down-regulation of E-cadherin, occludin and claudins that maintain cell tight junctions, and the up-regulation of N-cadherin, MMPs, vimentin and fibronectin that are essential for promoting motility and invasiveness [259]. Two major signaling pathways that induce EMT *via* these effectors are the TGF-β and Wnt pathways, both of which have been demonstrated to be regulated by iron chelation [156, 260] (Figure 5).

The role of TGF-β signaling in cancer is complex and paradoxical. In contrast to canonical tumor-suppressive TGF-β signaling in normal epithelial cells (described above), non-canonical TGF-β signaling is known to play important roles in promoting tumor progression and metastasis *via* induction of the EMT, stimulating cell proliferation and evasion of the immune system [259, 261]. This effect probably results from progressive mutations that collectively inactivate the core elements of the TGF-β signaling cascade and its downstream effectors, allowing tumor cells to escape the tumor-suppressive mechanisms of TGF-β and activate oncogenic signaling pathways [259]. In fact, TGF-β stimulation has been observed to induce cell proliferation *via* the Ras/Raf/MEK/ERK pathway, rather than suppress it *via* normal SMAD signaling, in TSU-Pr1 prostate cancer cells [262]. SMAD-dependent TGF-β signaling promotes EMT by activating the mesenchymal-associated transcription factors, Snail, Slug, Twist and ZEB1, amongst others [263–265]. Further, SMADs also up-regulate cytoskeleton-associated genes that mediate cell motility and invasion, including vimentin and fibronectin [266].

In addition to activating the trimeric SMAD complex, TGF-β can also activate the MAPK, Rho/Rho-associated, coiled-coil containing protein kinase 1 (ROCK1) and PI3K pathways, amongst others [261, 267] (Figure 5). Each of these pathways can also contribute to the EMT. For example, ERK MAPK signaling enhances repression of E-cadherin and activates Twist, N-cadherin and MMP expression [268, 269]. TGF-β-mediated activation of PI3K/AKT signaling induces activation of mammalian target of rapamycin complexes 1/2 (mTORC1/2) that aids the EMT, increasing expression of Snail and MMP-9, and repressing E-cadherin expression [270, 271]. Phosphorylation and inhibition of GSK3β that is regulated by AKT also stabilizes Snail and Slug expression [272, 273]. Moreover, TGF-β can also induce the activation of Rho-like GTPases, *e.g*., RhoA, that then activates the ROCK1 and LIM kinase (LIMK) pathways to drive dynamic actin reorganization, lamellipodia and filopodia formation required for the EMT [274, 275] (Figure 5).

Another key regulator of EMT is the Wnt signaling pathway that has canonical roles in development, tumorigenesis and metastatic progression [276]. It is activated at the plasma membrane by binding of Wnt ligands to its co-receptors, Frizzled and LDL receptor-related protein 5/6 (LRP5/6) [277]. In the absence of Wnt signaling, the destruction complex, which is composed of adenomatous polyposis coli (APC), axin and GSK3β, phosphorylates β-catenin that then promotes its proteasomal degradation [276] (Figure 5). However, in the presence of Wnt ligands, this complex disintegrates, stabilizing free β-catenin in the cytosol and allowing its nuclear translocation where it interacts with T-cell factor (TCF)/lymphoid enhancer-binding factor (LEF) transcription factors to promote transcription of pro-metastatic genes [276]. Wnt signaling also inhibits GSK3β-mediated degradation of the mesenchymal transcription factors, Snail and Slug [278, 279] (Figure 5).

Recent studies have identified important roles for cellular iron in regulating the EMT that occur *via* iron chelator-mediated up-regulation of the metastasis suppressor, NDRG1 [146, 147]. In DU145 prostate and HT29 colon cancer cells, the chelators DFO and Dp44mT attenuated TGF-β-induced loss of E-cadherin and β-catenin at the cell membrane (Figure 5), and also decreased TGF-β-induced up-regulation of vimentin [156]. This demonstrated that these iron chelators could inhibit TGF-β-induced EMT in these cells. Moreover, NDRG1 expression, which is markedly up-regulated by these compounds, also inhibited the TGF-β-induced EMT in DU145 and HT29 cells [156]. Specifically, NDRG1 knockdown in these cells assumed a spindle-shaped morphology, abolished expression of E-cadherin and β-catenin at the membrane, and increased nuclear expression of β-catenin [156]. NDRG1 over-expression in DU145 and HT29 cells attenuated the ability of TGF-β to induce changes to morphology and expression of EMT markers that are characteristic of the EMT, as well as the migratory and invasive potential of these cells [156]. This effect was found to involve the transcriptional E-cadherin repressors, Snail and Slug. Similar outcomes from NDRG1 inhibiting the EMT were also reported in oral squamous cell carcinoma cells through up-regulation of E-cadherin and down-regulation of N-cadherin, vimentin, Snail, Slug, MMP-2 and MMP-9 [280]. Further studies revealed that NDRG1 inhibits β-catenin phosphorylation and nuclear translocation, specifically by up-regulating FRAT1, which prevents GSK3β from binding the β-catenin destruction complex [281]. NDRG1 could also inhibit β-catenin nuclear translocation that is mediated by Wnt signaling *via* reducing nuclear localization of p21 activated kinase 4 (PAK4) [281]. Together, these NDRG1-mediated effects lead to increased levels of β-catenin that is expressed at the cell membrane, where it functions to promote cell adhesion, while inhibiting its localization in the cell nucleus, where it is oncogenic [281, 282].

Studies performed in PC3mm metastatic prostate and MCF7 breast cancer cells demonstrated that NDRG1 interacts with the Wnt receptor, LRP6, subsequently blocking the Wnt cascade to suppress the EMT and metastasis by reactivating GSK3β, promoting β-catenin degradation, and inhibiting downstream expression of ATF3 [107]. Moreover, these studies showed that Dp44mT could significantly enhance NDRG1 expression in human breast cancer cells and suppress bone metastasis *in vivo* [107]. This observation indicated that Dp44mT-mediated NDRG1 up-regulation could play a critical role in negatively regulating Wnt signaling during EMT and metastasis (Figure 5).

Multiple iron chelators, including DFO, deferasirox and a series of acyl hydrazones have been shown to destabilize β-catenin, subsequently inhibiting Wnt signaling to inhibit colorectal cancer cell growth [260]. These agents also decreased the expression of Wnt target genes, which was shown to occur *via* β-catenin degradation downstream of the destruction complex. Excess iron levels have also been implicated in colorectal tumorigenesis through enhancement of Wnt signaling in cells displaying APC loss or abnormal β-catenin expression [283]. This effect increased cellular proliferation, correlating with increased mRNA expression of its targets, *c-myc* and *Naked cuticle 1* (*Nkd1*). Collectively, these studies identify important roles for iron in the regulation of multiple EMT mediators that could be exploited in cancer therapy using potent iron chelators.

Interestingly, DFO has also been reported to promote the EMT and enhance cell migration and invasion in HT29 cells, which was suggested to be mediated by DFO-induced HIF-1 expression [284]. However, this is in contrast with multiple reports previously described indicating that DFO exhibits inhibitory effects on tumor growth and the EMT [5, 156, 260], possibly reflecting differences in experimental conditions utilized in the study.

### Migration and metastasis

In addition to regulating the EMT, directed cell invasion and migration of tumor cells is required for metastasis [285]. This process involves multiple events, including F-actin polymerization, its interaction with myosin II filaments, and the subsequent formation of contractile actomyosin structures such as stress fibers [286]. These are rearranged and extended within the cell in response to various signaling cues to promote cytoskeletal reorganization and cell motility [287]. The small Rho-GTPase family is involved in regulating stress fiber assembly, contraction and motility through effectors such as Rho and Rac [288]. These molecules can subsequently regulate multiple targets to regulate the actin cytoskeleton, including the p21 activated kinase (PAK), ROCK1/myosin light chain (MLC) and LIMK pathways that mediate the formation and contractility of stress fibers [289], of which phosphorylated MLC is a key mediator of actin filament polymerization, stress fiber assembly and cell migration [290].

We recently demonstrated that iron chelation could inhibit cell migration and metastasis by modulating the ROCK1/MLC2 cascade [157] (Figure 5). Specifically, DFO, Dp44mT and DpC decreased the expression of ROCK1 and phosphorylated MLC2 in DU145, HT29 and HCT116 cells [157]. Importantly, the observed effects of these iron chelators was likely mediated *via* the ability of these agents to up-regulate NDRG1, since suppression of endogenous NDRG1 expression at least partially attenuated the ability of the chelators to inhibit ROCK1 expression, MLC2 phosphorylation and migration [157]. These studies elucidated the inhibitory role of NDRG1 on the ROCK1/MLC2 pathway, with NDRG1 over-expression inhibiting F-actin polymerization and stress fiber formation through regulation of this cascade [157].

To further understand the role of NDRG1 in cellular migration, its effects on the oncogene, c-Src, were important to investigate as the downstream effectors of this molecule play critical roles in regulating this process [291, 292]. Importantly, NDRG1 inhibited Src-induced phosphorylation of p130Cas and prevented its complex formation with CrkII [293]. In addition, NDRG1 could decrease activation of c-Abl and subsequent CrkII phosphorylation [293]. Collectively, this resulted in the inhibition of Rac1 signaling and decreased levels of downstream phosphorylated PAK1, playing critical roles in cytoskeletal dynamics and the regulation of cell motility [294]. Given that the iron chelators, DFO, Dp44mT and DpC, have been demonstrated to inhibit Src and c-Abl activity in certain cancer cell-types [121] and that they can markedly up-regulate NDRG1, it is plausible that these compounds could exhibit their anti-metastatic effects *via* NDRG1-mediated modulation of these pathways.

### ER stress and autophagy

**Figure 6:**
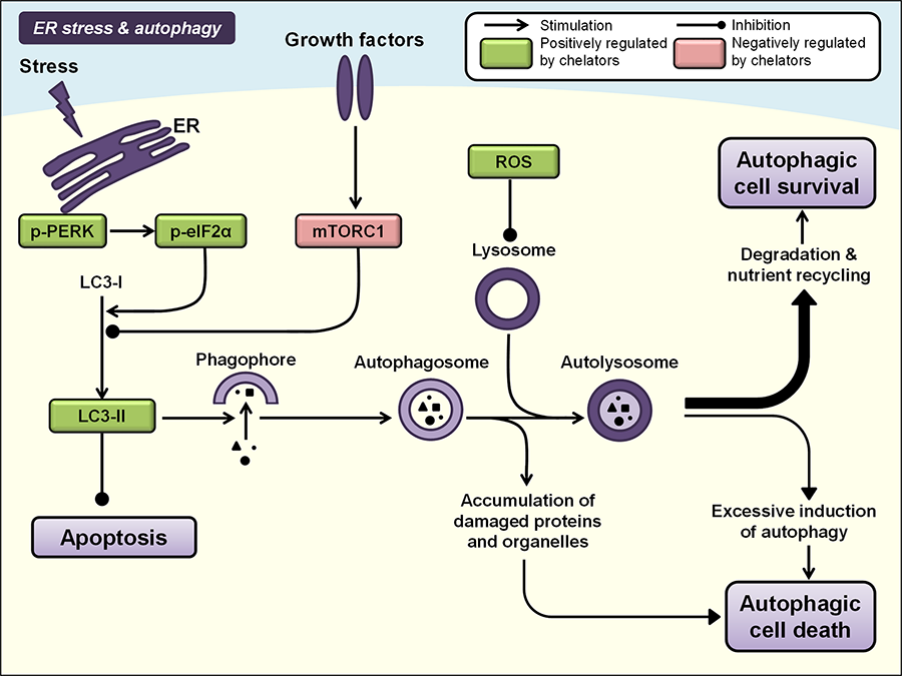
Iron chelation modulates ER stress and autophagy pathways Autophagy involves the formation of double-membrane vesicles from phagophores, *i.e*., autophagosomes, which engulf cytoplasmic contents and organelles. Fusion of the autophagosome with a lysosome forms an autolysosome that results in the degradation of its contents, nutrient recycling and autophagic cell survival. However, prolonged autophagy in certain settings can also lead to non-apoptotic autophagic cell death. Two core regulators of autophagy include the PERK/eIF2α pathway that is activated by endoplasmic reticulum (ER) stress, and the PI3K/AKT/mTORC1 pathway. Iron chelators have been demonstrated to modulate both these pathways and subsequent autophagic processes, and also enhance expression of LC3-II, which is recruited to autophagosomal membranes during induction of autophagy (see text for more details). Molecules negatively regulated by iron chelators are indicated by red boxes, and those positively regulated are indicated by green boxes.

Autophagy refers to a homeostatic, catabolic process that degrades cellular proteins and organelles to sustain cellular metabolism under conditions of stress [295]. It is initiated by the formation of crescent-shaped structures called phagophores that surround targeted cytoplasmic constituents [296] (Figure 6). Concomitantly, lipid-bound microtubule-associated protein 1A/1B-light chain 3 (LC3) is converted from its soluble form (LC3-I) to its lipid-bound form (LC3-II) and is expressed on the membrane of phagophores, subsequently leading to the formation of double-membrane vesicles, *i.e*., autophagosomes, which engulf the target cargo [296]. The autophagosome subsequently matures by fusing with a lysosome to form an autolysosome, exposing the cargo to lysosomal hydrolases that promote degradation of its contents and nutrient recycling [297] (Figure 6).

Complex signaling networks control mammalian autophagy, with the integration of stress signals through the PI3K/AKT/mTORC1 pathway acting as a core negative regulator of the autophagic machinery [297]. Activation of the PI3K/AKT signaling cascade inhibits TSC2 [174], activating the mTORC1 complex and inhibiting formation of a trimeric unc-51-like kinase (ULK) complex required for autophagosome formation from the phagophore [298]. Thus, mTORC1 activity inhibits autophagic initiation (Figure 6). Another key regulator of autophagy is adenosine monophosphate kinase (AMPK), which acts as a major stimulator of autophagy through its ability to activate TSC1/2 and suppress mTORC1 signaling [298–300], but also potentially through direct phosphorylation of ULK [301].

Activation of the endoplasmic reticulum (ER) stress pathway and unfolded protein response can also potently induce autophagy [299]. Specifically, the binding of accumulated unfolded proteins to the ER chaperone Grp78/Bip induces the release of three ER membrane-associated proteins that trigger distinct pathways, namely protein kinase-like endoplasmic reticulum kinase (PERK), activating transcription factor 6 (ATF6) and inositol-requiring enzyme 1 (IRE1) [302]. Phosphorylation and activation of PERK directly induces phosphorylation of eukaryotic initiation factor 2α (eIF2α) that is critical for the transcription of key autophagy-associated genes during ER stress, mediating LC3 conversion and subsequent autophagosome formation [303] (Figure 6).

Autophagy serves dual roles in tumorigenesis and tumor progression [299]. Since defects in autophagy have been linked with susceptibility to genomic damage and tumorigenesis in mice [304], autophagy could suppress tumorigenesis by limiting cell growth and genomic instability [305]. However, autophagy can also maintain the survival of tumor cells, allowing adaptation to stress conditions induced by high metabolic demands, hypoxic tumor microenvironments or chemotherapeutics [299]. In human pancreatic adenocarcinoma cell lines and tumor specimens, high levels of basal autophagy allowed the maintenance of cellular energy production that fuelled tumor growth [306]. Moreover, inhibiting autophagy in these cells by genetic means or chloroquine treatment promoted tumor regression of xenografts *in vivo* and mice survival [306]. Indeed, this has formed the underlying basis for preclinical and ongoing clinical studies that are administering autophagy inhibitors, *e.g*., chloroquine and hydroxychloroquine, in combination with chemotherapy [299].

However, prolonged autophagy in certain cellular settings can overwhelm the capacity of the cell, leading to a non-apoptotic pro-death pathway known as autophagic cell death [307]. Thus, targeting autophagy for cancer treatment presents as a “double-edged sword”, with its role in tumor progression and treatment response remaining incompletely understood. Harnessing this pro-death cascade of the autophagic process could be a novel vantage point of attack against tumor cells already existing under conditions of high stress.

Recently, it was demonstrated that Dp44mT modulates the autophagic process to favor cell death in breast cancer cells by increasing autophagosome synthesis, but also inhibiting subsequent autolysosome formation by impairing lysosomal integrity [308]. This illustrates a unique mechanism by which the double-edged autophagic machinery can be exploited in cancer therapy. Specifically, Dp44mT induced autophagic initiation as demonstrated by increased LC3-II levels, and also increased expression of another autophagy marker, namely p62, due to suppressed autophagosome degradation [308]. This latter effect was found to be dependent on the ability of Dp44mT to generate ROS, which subsequently permeate and destabilize the lysosome, preventing autolysosome formation and autophagosome degradation [308]. Thus, rather than enabling completion of the autophagic process that typically promotes cancer cell survival through nutrient recycling, the accumulation of damaged proteins and organelles triggers cell death [308] (Figure 6). These studies also postulated that DFO inhibited autophagosome degradation, but did not increase autophagosome synthesis, and thus, led to LC3-II accumulation in these cells [308].

The chelators, DFO and Dp44mT, also induced autophagy in PANC-1 pancreatic cancer cells as demonstrated by increased LC3-II expression [309]. This occurred *via* induction of ER stress and activation of the PERK/eIF2α signaling cascade, as demonstrated by increased levels of p-PERK and p-eIF2α [309]. The ability of these compounds to sequester cellular iron was critical for its effects on autophagy induction, as incubation with the DFO:Fe complex or Dp2mT did not exhibit the same effects [309]. Incubation with iron- or copper-complexed Dp44mT also elevated LC3-II expression, which was attenuated by NAC, indicating that Dp44mT-mediated redox activity is important for its role in regulating LC3-II expression [309]. Interestingly, NDRG1 was found to suppress the stress-induced autophagic response, which could mediate the anti-tumor and anti-metastatic activities of NDRG1 by enhancing apoptosis and inhibiting metastasis [309]. This was shown to occur *via* inhibition of the PERK/eIF2α pathway involved in the unfolded protein response [309].

Given that Dp44mT markedly up-regulates NDRG1, the seemingly opposing effects of Dp44mT and NDRG1 on autophagy appear contradictory [309]. However, the initial Dp44mT-mediated activation of autophagic initiation is likely to be subsequently suppressed due to up-regulation of NDRG1 following longer periods of exposure to Dp44mT [309]. This is consistent with the notion that NDRG1 acts in response to cellular stress [148, 310], which could lead to suppression of stress-induced autophagy [309]. Further, in addition to the generation of ROS by Dp44mT that causes lysosomal membrane permeabilization that disturbs the autophagic process [308], it is important to note that Dp44mT affects multiple cellular targets to induce its anti-tumor activity, as described herein and elsewhere [5].

Autophagy has also been implicated in the anti-tumor mechanism of DFO and deferasirox in multiple myeloma cell lines [311]. Specifically, exposure of myeloma cell lines to DFO and deferasirox markedly induced LC3-II expression concomitant with induction of autophagy, and the autophagy inhibitor 3-methyladenine rescued these cells from chelator-induced cytotoxicity [311]. This was potentially mediated by the suppressive effect of these compounds on the mTORC1 substrate p70S6 kinase, which negatively regulates autophagy [312]. Moreover, DFO was shown to induce autophagy flux and decrease caspase activation, inhibiting TRAIL-mediated apoptosis, in colon cancer cells [313].

Of importance, the iron storage protein, ferritin, is reported to be degraded in the cell *via* autophagy [314, 315]. Multiple iron chelators can direct the pathway *via* which ferritin is degraded in cells [316]. In these studies, incubation of cells with poorly permeable DFO resulted in lysosome-mediated degradation of ferritin *via* autophagy [316]. The smaller, more lipophilic and membrane-permeable ligands, deferasirox and deferiprone, diverted ferritin degradation towards the proteasomal pathway [316]. This contrasted with previous findings [311], which may be explained by cell-type-dependent differences in the regulation of autophagic processing. Moreover, considering that autophagy is a dynamic process [317], and that these latter studies only visualized LC3-II-containing autophagosomes as a autophagic marker [311, 316], further studies utilizing late-stage autophagy inhibitors, such as bafilomycin A1 or choloroquine, in combination with iron chelators are necessary to determine whether the increased number of autophagosomes observed are due to enhanced autophagic initiation or suppression of lysosomal-mediated autophagic degradation [317]. Collectively, these recent studies clearly identify induction of autophagy as a potential mediator of cytotoxic iron chelators, requiring further elucidation of the precise mechanisms responsible to aid its clinical application (Figure 6).

## CONCLUSIONS AND FUTURE DIRECTIONS

Compelling evidence exists to support the preclinical and clinical development of DpT thiosemicarbazones as anti-cancer agents. Moreover, these compounds demonstrate synergistic activities with existing chemotherapies including gemcitabine, cisplatin, doxorubicin and tamoxifen [113, 318]. Thus, these compounds could be effectively utilized in combination cancer therapies.

Major advances have been achieved in recent years in terms of gaining mechanistic insight into how the DpT ligands exert their multi-modal effects at the molecular level by targeting oncogenic signaling pathways. This is a critical step in the development of these compounds, providing valuable information related to their potential application as multi-targeted therapies based on the genetic profiles and stages of individual tumors. However, differences between cancer cell-types as well as complexities in signaling pathway crosstalk and feedback loops mean that the molecular mechanisms of DpT thiosemicarbazones still require careful elucidation. The studies described herein will no doubt provide a solid foundation for future studies and provide necessary justification for the planned clinical trials in humans.
